# Predominant CD8^+^ cell infiltration and low accumulation of regulatory T cells in immune checkpoint inhibitor‐induced tubulointerstitial nephritis

**DOI:** 10.1111/pin.13428

**Published:** 2024-04-18

**Authors:** Kenta Tominaga, Etsuko Toda, Kazuhiro Takeuchi, Shoichiro Takakuma, Emi Sakamoto, Hideaki Kuno, Yusuke Kajimoto, Yasuhiro Terasaki, Shinobu Kunugi, Akiko Mii, Yukinao Sakai, Mika Terasaki, Akira Shimizu

**Affiliations:** ^1^ Department of Analytic Human Pathology Nippon Medical School Bunkyo‐ku Tokyo Japan; ^2^ Division of Nephrology and Hypertension The Jikei University School of Medicine Minato‐ku Tokyo Japan; ^3^ Division of Pathology Nippon Medical School Hospital Bunkyo‐ku Tokyo Japan; ^4^ Department of Nephrology Nippon Medical School Musashi Kosugi Hospital Kawasaki‐shi Kanagawa Japan; ^5^ Department of Endocrinology, Metabolism and Nephrology Nippon Medical School Bunkyo‐ku Tokyo Japan

**Keywords:** immune checkpoint inhibitor, regulatory T cells, tubulointerstitial nephritis

## Abstract

Immune checkpoint inhibitors (ICIs) can provide survival benefits to cancer patients; however, they sometimes result in the development of renal immune‐related adverse events (irAEs). Tubulointerstitial nephritis (TIN) is the most representative pathological feature of renal irAEs. However, the clinicopathological entity and underlying pathogenesis of ICI‐induced TIN are unclear. Therefore, we compared the clinical and histological features of this condition with those of non‐ICI drug‐induced TIN. Age and C‐reactive protein levels were significantly higher in ICI‐induced TIN, but there were no significant differences in renal function. Immunophenotyping of ICI‐induced TIN showed massive T cell and macrophage infiltration with fewer B cells, plasma cells, neutrophils, and eosinophils. Compared with those in non‐ICI drug‐induced TIN, CD4^+^ cell numbers were significantly lower in ICI‐induced TIN but CD8^+^ cell numbers were not significantly different. However, CD8/CD3 and CD8/CD4 ratios were higher in ICI‐induced TIN. Moreover, CD25^+^ and FOXP3^+^ cells, namely regulatory T cells, were less abundant in ICI‐induced TIN. In conclusion, T cell, B cell, plasma cell, neutrophil, and eosinophil numbers proved useful for differentiating ICI‐induced and non‐ICI drug‐induced TIN. Furthermore, the predominant distribution of CD8^+^ cells and low accumulation of regulatory T cells might be associated with ICI‐induced TIN development.

**Abbreviations**
AKI: acute kidney injury
BPH: benign prostatic hyperplasia
CI: cerebral infarction
CRP: C‐reactive protein
CTLA‐4: anticytotoxic T‐lymphocyte‐associated protein 4
DL: dyslipidemia
DM: diabetes mellitus
HT: hypertension
HUA: hyperuricemia
IBS: irritable bowel syndrome
ICI: immune checkpoint inhibitors
IP: interstitial pneumonia
irAE: renal immune‐related adverse event
LC: liver cirrhosis
PD1: antiprogrammed cell death 1
PD‐L1: antiprogrammed death‐ligand 1
sCr: serum creatinine
TCR: T cell receptor
TIN: tubulointerstitial nephritis
Tregs: regulatory T cells

## INTRODUCTION

Immune checkpoint inhibitors (ICIs) can contribute significantly to the treatment of many malignancies, including metastatic melanoma.^1^ There are three main types of ICIs—antiprogrammed cell death 1 (PD1), antiprogrammed death‐ligand 1 (PD‐L1), and anticytotoxic T‐lymphocyte‐associated protein 4 (CTLA‐4) antibodies. Anti‐PD1 and anti‐PD‐L1 antibodies block the PD‐1/PD‐L1 pathway and induce immune activity against tumors^2^ whereas anti‐CTLA‐4 antibodies block the CTLA‐4/B7 (CD80/CD86) pathway in T cells, leading to the disruption of inhibitory immune signaling.^3^

ICIs can activate T cells and exert antitumor effects by blocking either pathway. However, they can also lead to the development of immune‐related adverse events (irAEs). Approximately 90% of patients undergoing ICI treatment experience irAEs of differing severity.^4^ These events can affect any organ, including the skin, gastrointestinal tract, liver, heart, thyroid, adrenal gland, pituitary gland, and kidneys.^5^ The most common pathological feature of renal irAEs is tubulointerstitial nephritis (TIN).^6^ Lymphocytes are predominant in the tubulointerstitium with ICI‐induced TIN,^7^ which is consistent with the mechanism underlying the effect of ICIs. Non‐ICI drug‐induced TIN is also mediated by lymphocytes^8^ and is indistinguishable from ICI‐induced TIN. However, the pathophysiology differs between the two TIN types, warranting different treatments.

We previously reported a case of ICI‐induced TIN and renal granulomatous vasculitis in which large numbers of CD8^+^ cells and no or few regulatory T cells (Tregs) infiltrated the kidneys.^9^ However, the clinicopathological entity and underlying pathogenesis of ICI‐induced TIN were unclear. In the present study, we performed a comparative analysis of ICI‐induced and non‐ICI drug‐induced TIN to decipher the clinicopathological characteristics of ICI‐induced TIN, including the infiltrating inflammatory cell phenotypes.

## MATERIALS AND METHODS

### Study setting and design

We included all patients diagnosed with ICI‐induced and non‐ICI drug‐induced TIN between December 2016 and November 2022. All diagnoses were based on clinicopathological information. The ICIs included were PD‐1 and CTLA‐4 inhibitors and their combination. The exclusion criteria were interstitial inflammation secondary to conditions, such as IgG4‐related disease, Sjogren's syndrome, sarcoidosis, bone marrow diseases, and antineutrophil cytoplasmic antibody‐associated vasculitis. Thirteen patients from four university hospitals, for whom renal biopsies were available, were included.

Baseline clinical and laboratory data and information on medications were collected from digital medical records. Laboratory data included baseline serum creatinine (sCr), C‐reactive protein (CRP), urine N‐acetyl‐β‐d‐glucosaminidase (NAG), and β2‐microglobulin (β2MG) levels and the urine protein–creatinine ratio at baseline. The study was conducted according to the Declaration of Helsinki and was approved by the Research Ethics Committee of the Nippon Medical School, Tokyo, Japan (M‐2022‐066). We obtained informed consent in the form of opt‐out through the Nippon Medical School website.

### Assessment of pathological features

TIN was diagnosed through light microscopy, immunohistochemistry, and electron microscopy of kidney biopsies. Renal interstitial inflammatory cell types were investigated using immunohistochemistry. For immunohistochemical analysis, slides were scanned using a virtual slide system (Olympus VS200), and the frequency of positive cells per unit area was determined using the QuPath software (Ver. 0.3.2). The pathological features of ICI‐induced TIN were confirmed and compared with those of non‐ICI drug‐induced TIN.

### Immunohistochemical staining and microscopic analysis

Inflammatory cell infiltrates were detected via immunohistochemical staining with antibodies against CD3 (T cells; rabbit polyclonal; DAKO), CD20 (B cells; mouse monoclonal; DAKO), CD68 (macrophages; mouse monoclonal; DAKO), CD138 (plasma cells; mouse monoclonal; DAKO), CD4 (rabbit monoclonal; Roche), CD8 (rabbit monoclonal; Roche), CD25 (mouse monoclonal; Cell Marque), and FOXP3 (rabbit polyclonal). Neutrophils and eosinophils were identified through hematoxylin and eosin (HE) staining and counted on a virtual slide.

### Immunofluorescence staining for CD4, FOXP3, and CD8

Interstitial CD4^+^ and FOXP3^+^ cells were analyzed with a tyramide signal amplification detection system (Opal™ 4‐color Manual IHC Kit, Cat# NEL810001KT; PerkinElmer Inc.). Opal 520, Opal 570, Opal 690 were used for CD4, FOXP3, and CD8, respectively. Nuclei were counterstained with 4′,6‐diamidino‐2‐phenylindole (DAPI). Fluorescence images were obtained using PhenoImager Mantra2™ quantitative pathology workstation (Akoya Biosciences), and the fluorophores and autofluorescence were spectrally unmixed using the inForm® image analysis software (Akoya Biosciences).

### Quantification of pathological findings

The number of immunohistochemically positive cells, eosinophils, and neutrophils per unit area (mm^2^) was evaluated using the QuPath software (Ver. 0.3.2).^10^ Digital image data were transmitted to the Qupath software. Thereafter, all renal cortex areas were selected using a polygon tool. Active inflammatory and nonactive inflammatory areas stained with HE were selected using a 500 × 500 µm ellipse, under a high‐power field. Finally, immunohistochemically positive cells were detected in each selected area using positive cell detection based on parameters shown in Supporting Information: Table 1. Simultaneously, eosinophils and neutrophils were analyzed in 1 mm^2^ area. For the score compartments of the intensity threshold parameters, “Cell: DAB OD mean” and “Nucleus: DAB OD mean” were used for cell membrane and nuclear staining, respectively.

### Statistical analysis

Statistical analyses were performed using GraphPad Prism 9 (GraphPad Software Inc.). Continuous data are presented as mean ± standard error of the mean. The Mann–Whitney *U* test was used to assess group differences. Significance was set at *p* < 0.05.

## RESULTS

### Patient characteristics

Thirteen patients with TIN, including seven with ICI‐induced and six with non‐ICI drug‐induced TIN, were investigated. Individual patient data are shown in Table 1, and the summary and statistics for each group are shown in Table 2. In the ICI‐induced TIN group, most patients were male (86%; 6/7), and the mean age was 67 years. Moreover, 71% (5/7) of the patients took proton pump inhibitors and/or nonsteroidal anti‐inflammatory drugs at the time of the kidney biopsy. The most common immunotherapy regimen was pembrolizumab (71%, 5/7); two patients received a combination of ipilimumab and nivolumab. Patients with ICI‐induced TIN were significantly older than those with non‐ICI drug‐induced TIN (ICI‐induced TIN vs. non‐ICI drug‐induced TIN, 67 ± 3 vs. 47 ± 9 years; *p* = 0.0122) and had higher CRP levels (ICI‐induced TIN vs. non‐ICI drug‐induced TIN, 8.5 ± 2.1 vs. 0.9 ± 0.3 mg/dL; *p* = 0.0332). Differences in other laboratory data were not significant. In the non‐ICI drug‐induced TIN group, the ratio of men to women was the same and the most common cause of TIN was antibiotic use (67%, 4/6).

**Table 1:** Clinical features.

	Age/Sex	Diagnosis	Clinical history	Offending drug or Immunotherapy regimen	Other potential nephrotoxins
ICI	65/Male	Melanoma	HT, DL	Nivolumab+Ipilimumab	Lafutidine, Bezafibrate
	66/Male	Lung cancer	HT, DL, HUA	Pembrolizumab	Esomeprazole, Febuxostat
	78/Male	Lung cancer	BPH, DL	Pembrolizumab+Cisplatin+Pemetrexed	Rabeprazole, Pitavastatin
	73/Male	Lung cancer	CI, IBS	Pembrolizumab+Cisplatin+Pemetrexed	Nil
	66/Female	Cecal cancer	DM, IP, LC	Pembrolizumab	Esomeprazole, Furosemide, Spironolactone, Alendronate
	52/Male	Lung cancer	Nil	Pembrolizumab+Pemetrexed	Vonoprazan, Mirtazapine
	72/Male	Lung cancer	Nil	Nivolumab+Ipilimumab	Lansoprazole, Loxoprofen, Pregabalin
Non‐ICI	53/Female	TIN	Asthma	Clarithromycin	Nil
	62/Male	AKI	Dilated cardiomyopathy	Tazobactam/Piperacillin	Enalapril, Furosemide, Spironolactone, Esomeprazole
	63/Male	AKI	Nil	Levofloxacin	Nil
	31/Female	TIN	Nil	Loxoprofen	Nil
	11/Female	TIN	Nil	Amoxicillin	Nil
	61/Male	TIN	DM, HT, DL, Thyroiditis	Bisoprolol	Irbesartan, Rosuvastatin, Voglibose

Abbreviations: AKI, acute kidney injury; BPH, benign prostatic hyperplasia; CI, cerebral infarction; DL, dyslipidemia; HT, hypertension; HUA, hyperuricemia; IBS, irritable bowel syndrome; ICI, immune checkpoint inhibitors; IP, interstitial pneumonia; Ipilimumab, anti‐cytotoxic T‐lymphocyte‐associated protein 4 (CTLA‐4) antibody; LC, liver cirrhosis; Nivolumab, Pembrolizumab, antiprogrammed cell death 1 (PD1) antibody; TIN, Tubulointerstitial nephritis.

**Table 2:** Baseline demographic data.

	ICI	Non‐ICI	*p* Value
Male gender, *n*(%)	6(86%)	3(50%)	0.2657
Age, year	67 ± 3^a^	47 ± 9	0.0122
sCr, mg/dL	3.0 ± 0.7	2.8 ± 0.7	0.8357
CRP, mg/dL	8.5 ± 2.1^a^	0.9 ± 0.3	0.0332
U‐β2MG, mg/L	15599 ± 6456	29416 ± 13754	0.366
U‐NAG, mg/L	22 ± 9	22 ± 4	0.4452
U‐Pro, g/gCr	0.8 ± 0.4	0.7 ± 0.1	0.1929

Abbreviations: CRP, C‐reactive protein; ICI, immune checkpoint inhibitor, sCr, serum creatinine; TIN, tubulointerstitial nephritis; U‐pro, urine protein creatinine ratio; U‐β2MG, urine β2‐microglobulin; Urine NAG, urine N‐Acetyl‐β‐D‐glucosaminidase.

^a^ Compared with non‐ICI; *p* < 0.05.

### Assessment of pathological features associated with inflammatory cell infiltration

**Figure 1:**
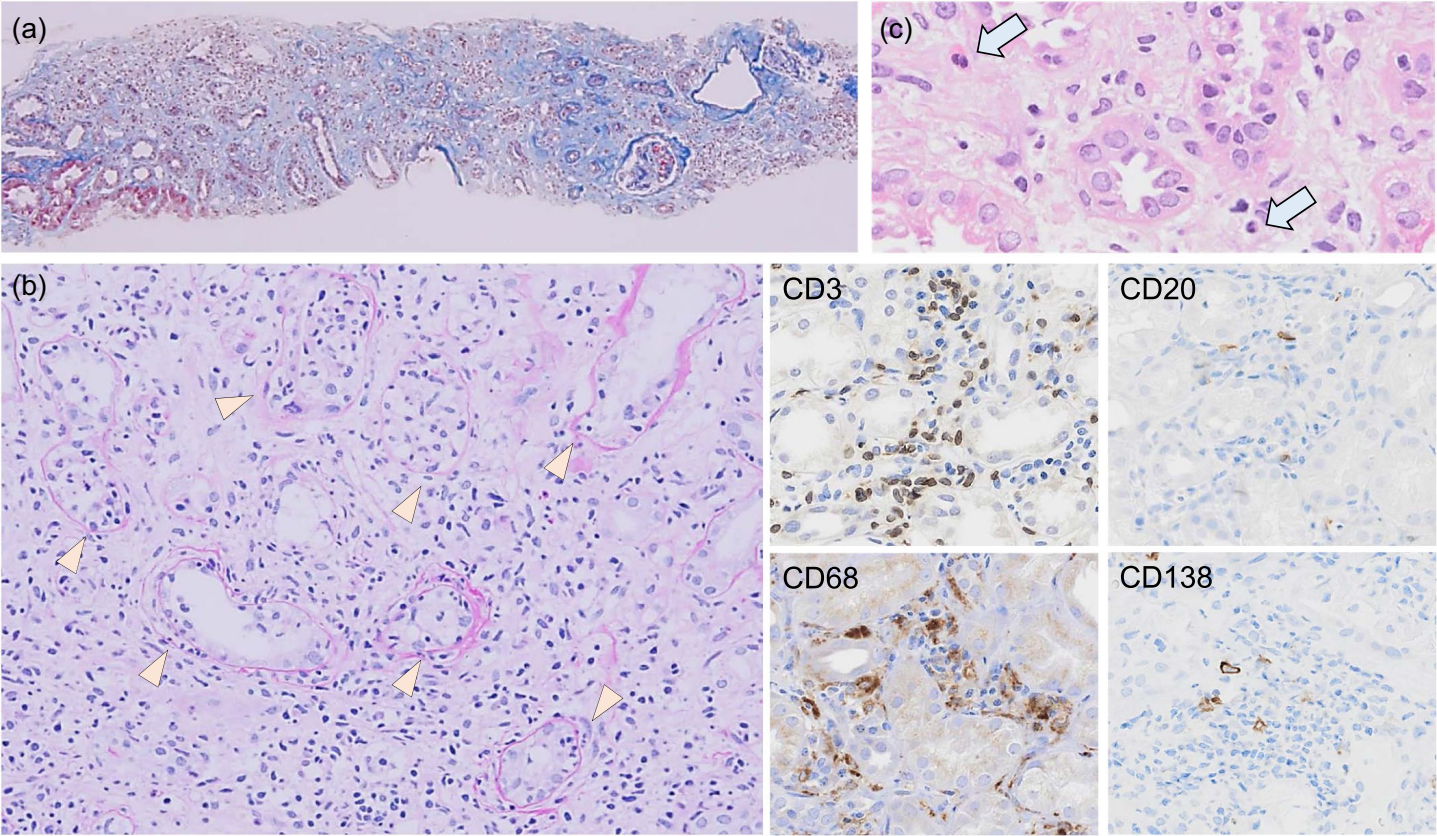
Infiltration of inflammatory cells in immune checkpoint inhibitor (ICI)‐induced tubulointerstitial nephritis (TIN). (a) Masson trichrome staining showing focal interstitial inflammatory cell infiltration with edematous fibrosis (×40). (b) Periodic acid‐Schiff (PAS) staining showing severe tubulitis with mononuclear tubulointerstitial infiltration (arrowhead, ×200). (c) Hematoxylin and eosin (HE) staining showing focal neutrophil and eosinophil infiltration in the interstitium (×200). Immunohistochemistry revealing predominant CD3^+^ and CD68^+^ cells with few CD20^+^ and CD138^+^ cells (×200).

**Figure 2:**
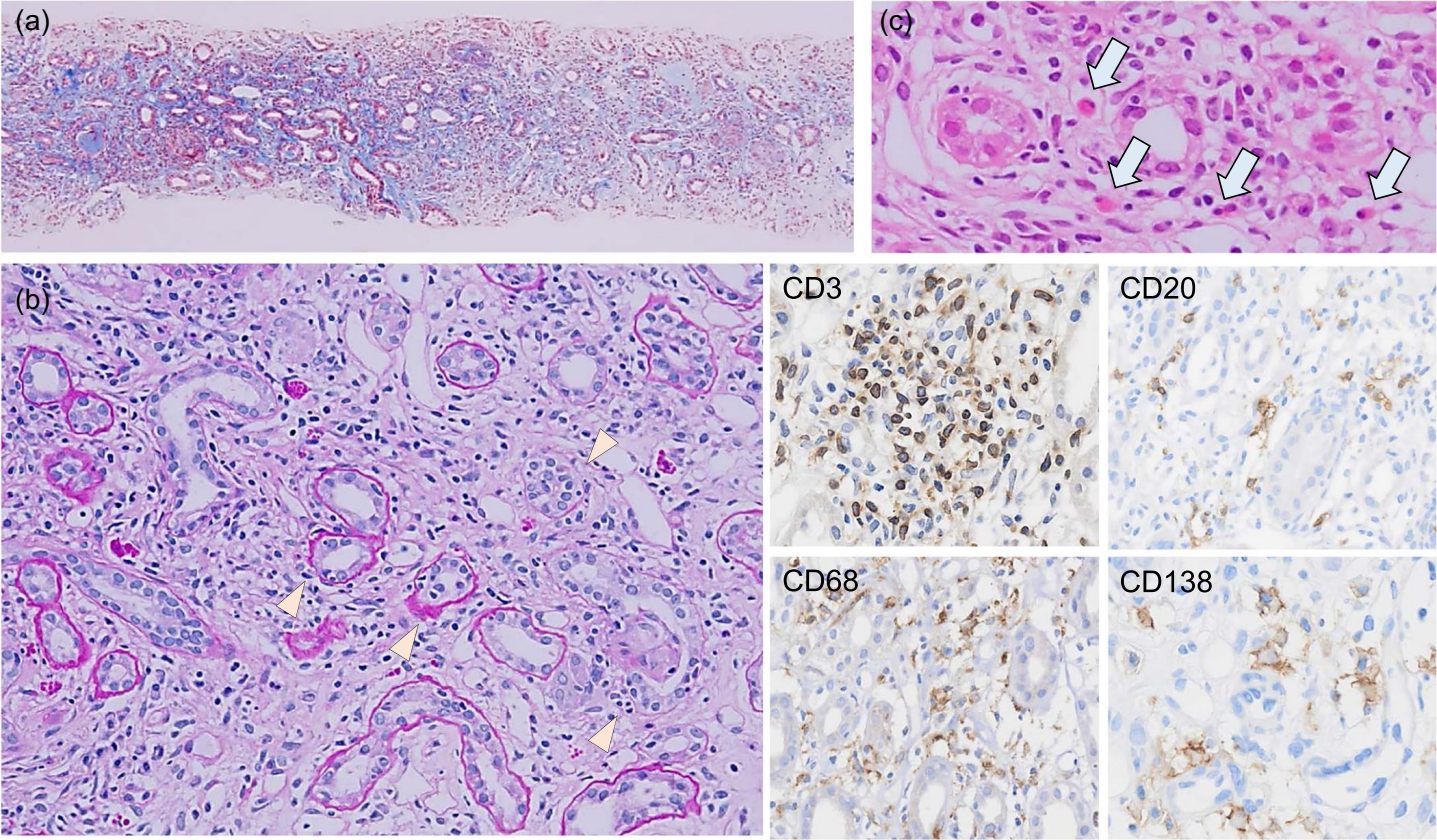
Infiltration of inflammatory cells in nonimmune checkpoint inhibitor (ICI) drug‐induced tubulointerstitial nephritis (TIN). (a) Masson trichrome staining showing diffuse interstitial inflammatory cell infiltration with edematous fibrosis (×40). (b) Periodic acid‐Schiff (PAS) staining showing moderate tubulitis with mononuclear tubulointerstitial infiltration (arrowhead, ×200). (c) Hematoxylin and eosin (HE) staining showing prominent eosinophil infiltration in the interstitium (×200). Immunohistochemistry revealing predominant CD3^+^ and CD68^+^ cells with focal tubulointerstitial CD20^+^ and CD138^+^ cell infiltration (×200).

The histological and immunohistochemical analyses of ICI‐induced TIN kidney biopsy specimens showed focal tubulointerstitial inflammatory cell infiltration and severe tubulitis with predominant T cell and macrophage infiltration, and few B cells, plasma cells, neutrophils, and eosinophils (Figure 1). However, renal biopsies from non‐ICI drug‐induced TIN were associated with focal moderate tubulitis and diffuse tubulointerstitial inflammation, with predominant T cell and macrophage infiltration, accompanied by focal tubulointerstitial B cell and plasma cell infiltration, as well as eosinophils (Figure 2). A comparative analysis of the number of inflammatory cells between the ICI‐induced and non‐ICI drug‐induced TIN groups was then performed (Table 3). The range of interstitial inflammatory cell infiltration was low in ICI‐induced TIN compared with that in non‐ICI drug‐induced TIN. Moreover, immunohistochemical staining showed that the number of infiltrating T cells, plasma cells, neutrophils, and eosinophils was significantly lower in ICI‐induced TIN (ICI‐induced TIN vs. non‐ICI drug‐induced TIN, range of interstitial inflammatory cell infiltration: 36 ± 12% vs. 83 ± 7%, *p* = 0.0216; T cells: 501 ± 125/mm^2^ vs. 1207 ± 146/mm^2^, *p* = 0.0047; plasma cells: 21 ± 8/mm^2^ vs. 194 ± 48/mm^2^, *p* = 0.0047; neutrophils: 6 ± 4/mm^2^ vs. 22 ± 7/mm^2^, *p* = 0.0221; eosinophils: 10 ± 6/mm^2^ vs. 69 ± 19/mm^2^, *p* = 0.0047).

**Table 3:** Pathological characteristics of inflammatory cell infiltration.

	ICI	Non‐ICI	*p* Value
Rate of inflammatory cell infiltration in the cortex(%)	36 ± 12^a^	83 ± 7	0.0216
Interstitial inflammatory cell counts per mm^2^			
T cells (CD3+ cells)	501 ± 125^a^	1207 ± 146	0.0047
B cells (CD20+ cells)	49 ± 15	172 ± 63	0.1014
Macrophages (CD68+ cells)	485 ± 197	746 ± 233	0.2949
Plasma cells (CD138+ cells)	21 ± 8^a^	194 ± 48	0.0047
Neutrophils	6 ± 4^a^	22 ± 7	0.0221
Eosinophils	10 ± 6^a^	69 ± 19	0.0047

Abbreviations: ICI, immune checkpoint inhibitor; TIN, tubulointerstitial nephritis.

^a^ Compared with non‐ICI; *p* < 0.05.

### Assessment of pathological features associated with inflammatory cell infiltration in active inflammatory areas

Next, we determined the differences in the number of inflammatory cells in ICI‐induced and non‐ICI drug‐induced TIN in active inflammatory areas due to the different rates of inflammatory cell infiltration in cortical areas (Supporting Information: Table 2). Immunohistochemical analysis showed that the number of infiltrating T cells, B cells, plasma cells, neutrophils, and eosinophils was significantly lower in ICI‐induced TIN (ICI‐induced TIN vs. non‐ICI drug‐induced TIN, T cells: 2137 ± 460/mm^2^ vs. 5493 ± 917/mm^2^, *p* = 0.014; B cells: 206 ± 88/mm^2^ vs. 769 ± 196/mm^2^, *p* = 0.035; plasma cells: 632 ± 399/mm^2^ vs. 2200 ± 729/mm^2^, *p* = 0.0198; neutrophils: 10 ± 5/mm^2^ vs. 41 ± 8/mm^2^, *p* = 0.0111; eosinophils: 11 ± 6/mm^2^ vs. 98 ± 49/mm^2^, *p* = 0.0087).

### Immunohistochemical analysis of T cell distribution

**Figure 3:**
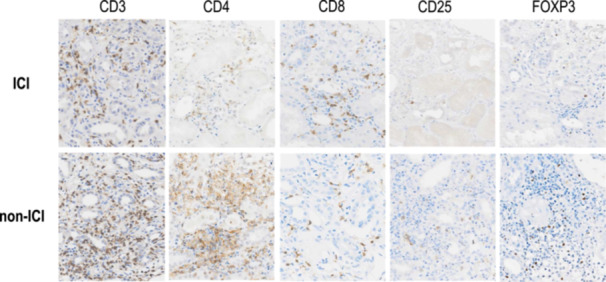
Infiltration of inflammatory cells in both immune checkpoint inhibitor (ICI)‐induced tubulointerstitial nephritis (TIN) and non‐ICI drug‐induced TIN. Representative images of immunohistochemical staining for CD3^+^, CD4^+^, CD8^+^, CD25^+^, and FOXP3^+^ cells in the renal cortex are shown; magnification, ×200.

**Figure 4:**
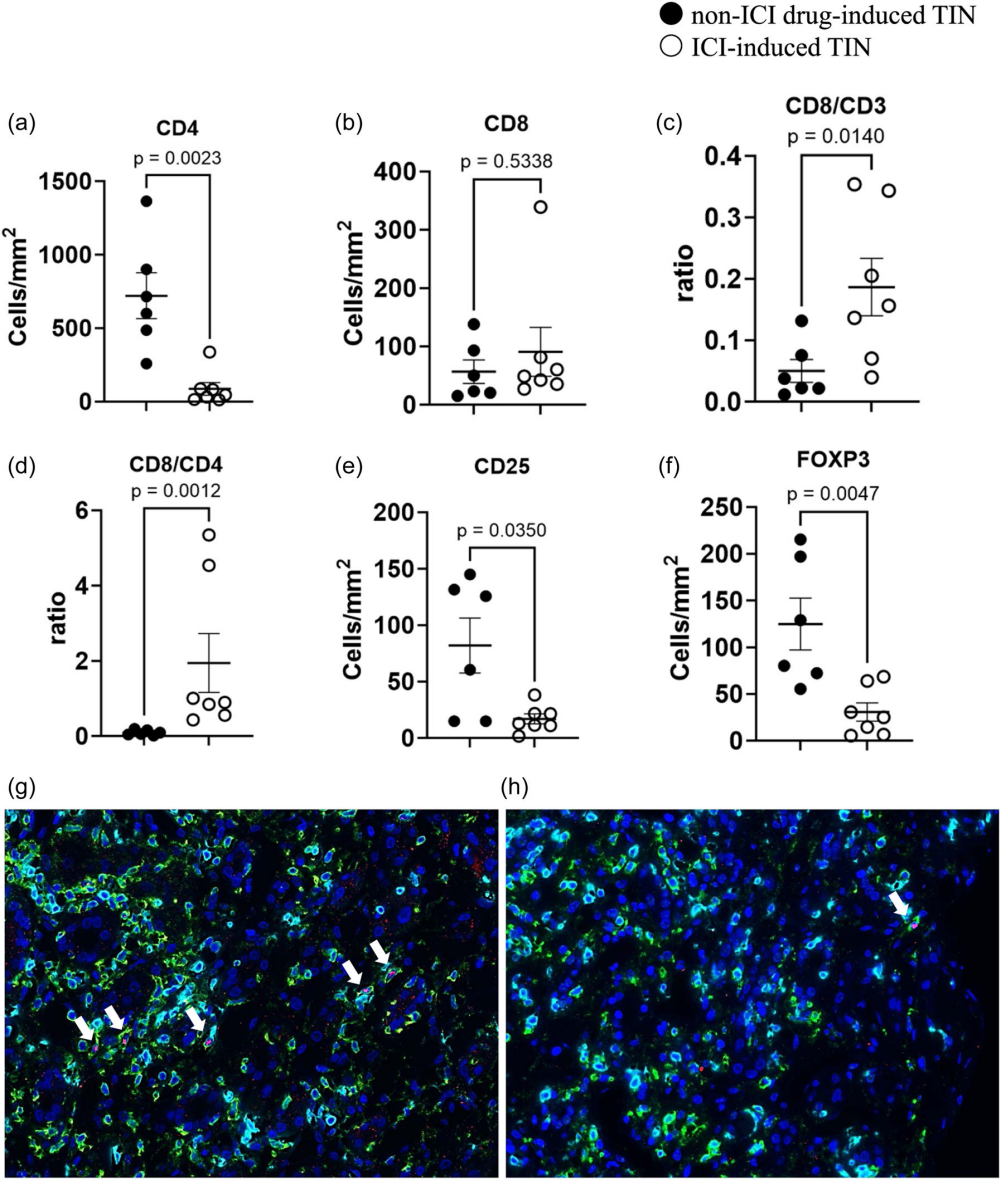
Immunohistochemical characterization of inflammatory cells. (a, b) Number of CD4^+^ and CD8^+^ cells per unit area (mm^2^) in the cortex in the two groups. (c, d) The CD8/CD3 and CD8/CD4 ratios in the two groups. (e, f) Numbers of CD25^+^ and FOXP3^+^ cells per unit area (mm^2^) in the cortex in the two groups. Results are presented as mean ± standard error of the mean, with the Mann–Whitney *U* test used to compare the two groups. Immunofluorescence staining of CD4^+^, FOXP3^+^ and CD8^+^cells and DAPI staining in ICI‐induced TIN and non‐ICI drug‐induced TIN. (g) Tubulointerstitial staining of CD4^+^ (green), FOXP3^+^ (red), CD8^+^ (clear blue), and DAPI (blue) in non‐ICI drug‐induced TIN is shown. Many CD4^+^ cells were observed in the tubulointerstitium, some of which were FOXP3^+^ with DAPI‐positive nuclei (white arrows). The number of CD8^+^ cells was fewer than that of CD4^+^ cells. (h) Predominant CD8^+^ cells (clear blue) are shown. Only one CD4^+^ (green), Foxp3^+^ (red), and DAPI (blue)‐positive cell (white arrow) is seen. ICI, immune checkpoint inhibitor; TIN, tubulointerstitial nephritis.

Representative immunohistochemical staining for CD3^+^, CD4^+^, CD8^+^, CD25^+^, and FOXP3^+^ cells is shown in Figure 3. A comparison of the data and immunofluorescence staining for CD4^+^, FOXP3^+^, and CD8^+^ cells in representative ICI‐induced TIN and non‐ICI drug‐induced TIN cases are shown in Figure 4. In patients with ICI‐induced TIN, the density of CD4^+^ cells was significantly lower than that in patients with non‐ICI drug‐induced TIN (ICI‐induced TIN vs. non‐ICI drug‐induced TIN, CD4: 88 ± 43/mm^2^ vs. 721 ± 156/mm^2^, *p* = 0.0023). Despite fewer CD3^+^ cells compared with that in non‐ICI‐induced TIN (Table 3), the density of CD8^+^ cells was not significantly different. Interestingly, the CD8/CD3 and CD8/CD4 ratios in patients with ICI‐induced TIN were higher than those in patients with non‐ICI drug‐induced TIN (ICI‐induced TIN vs. non‐ICI drug‐induced TIN, CD8/CD3: 0.19 ± 0.05 vs. 0.05 ± 0.02, *p* = 0.0140; CD8/CD4: 1.95 ± 0.78 vs. 0.09 ± 0.03, *p* = 0.0012). Moreover, the densities of CD25^+^ and FOXP3^+^ cells, specifically indicative of regulatory T cells, were lower in patients with ICI drug‐induced TIN (ICI‐induced TIN vs. non‐ICI drug‐induced TIN, CD25: 17 ± 4/mm^2^ vs. 82 ± 24/mm^2^, *p* = 0.0350; FOXP3: 31 ± 10/mm^2^ vs. 125 ± 28/mm^2^, *p* = 0.0047).

### Comparison of T cell distribution in active inflammatory areas between ICI‐induced and non‐ICI drug‐induced TIN

T cell distribution in active inflammatory areas in ICI‐induced and non‐ICI drug‐induced TIN (Supporting Information: Figure 1) was similar to the overall distribution (Figure 4). Significant differences were noted in the density of CD4^+^, CD25^+^, and FOXP3 cells (ICI‐induced TIN vs. non‐ICI drug‐induced TIN, CD4: 414 ± 74/mm^2^ vs. 3193 ± 535/mm^2^, *p* = 0.0006; CD25: 44 ± 13/mm^2^ vs. 485 ± 212/mm^2^, *p* = 0.0256; FOXP3: 102 ± 44/mm^2^ vs. 493 ± 49/mm^2^, *p* = 0.0017). However, the density of CD8^+^ cells was not significantly different. On the contrary, the CD8/CD3 and CD8/CD4 ratios in patients with ICI‐induced TIN were higher than those in patients with non‐ICI drug‐induced TIN (ICI‐induced TIN vs. non‐ICI drug‐induced TIN, CD8/CD3: 0.2 ± 0.07 vs. 0.04 ± 0.02, *p* = 0.0140; CD8/CD4: 0.7 ± 0.17 vs. 0.1 ± 0.06, *p* = 0.0017).

### T cell distribution in nonactive inflammatory areas of ICI‐induced TIN compared with that in active inflammatory areas of ICI‐induced TIN and non‐ICI drug‐induced TIN

We also compared nonactive inflammatory areas with active inflammatory areas in ICI‐induced TIN and the overall area in non‐ICI drug‐induced TIN that showed a uniform inflammation area with regard to T cell distribution (Supporting Information: Table 3). The CD8/CD3, CD8/CD4, FOXP3/CD4, and FOXP3/CD8 ratios were not significantly different between nonactive and active inflammatory areas in ICI‐induced TIN. Although there was no significant difference in the CD8/CD3, CD8/CD4, and FOXP3/CD4 ratios, the FOXP3/CD8 ratio was significantly different in nonactive inflammatory areas in ICI‐induced TIN and non‐ICI drug‐induced TIN (nonactive inflammatory areas in ICI‐induced TIN vs. non‐ICI drug‐induced TIN, FOXP3/CD8: 0.4 ± 0.1 vs. 4.2 ± 2.0, *p* = 0.0023). The CD8/CD3 and CD8/CD4 ratios were significantly increased, whereas the FOXP3/CD8 ratio was significantly decreased in active inflammatory areas in ICI‐induced TIN compared with that in nonactive inflammatory areas in non‐ICI drug‐induced TIN (active inflammatory areas in ICI‐induced TIN vs. non‐ICI drug‐induced TIN, CD8/CD3: 0.2 ± 0.1 vs. 0.05 ± 0.02, *p* = 0.035; CD8/CD4: 0.7 ± 0.2 vs. 0.09 ± 0.03, *p* = 0.0047; FOXP3/CD8: 0.4 ± 0.1 vs. 4.2 ± 2.0, *p* = 0.0012).

## DISCUSSION

A representative renal biopsy of ICI‐induced TIN showed focal tubulointerstitial inflammatory cell infiltration with moderate‐to‐severe tubulitis. The infiltrates predominantly comprised T cells and macrophages, with few B cells, plasma cells, neutrophils, and eosinophils. One feature that can be used to differentiate non‐ICI drug‐induced TIN is that ICI‐induced TIN has fewer T cells, plasma cells, neutrophils, and eosinophils in the tubulointerstitium. By comparing ICI‐induced and non‐ICI drug‐induced TIN through an immunohistochemical analysis of the distribution of T cells, we demonstrated that in ICI‐induced TIN, regardless of the activity of inflammation, CD8^+^ cells were predominant among T cells, with fewer cells positive for CD25 and FOXP3 (characteristic markers of Tregs). In addition, although the CD8/CD3, CD8/CD4, FOXP3/CD4, and FOXP3/CD8 ratios in nonactive inflammatory areas and active inflammatory areas in ICI‐induced TIN were not significantly different, the FOXP3/CD8 ratio in nonactive inflammatory areas in ICI‐induced TIN was significantly lower than that in non‐ICI drug‐induced TIN. To our knowledge, this is the first study to suggest that the predominant CD8^+^ cell distribution and low accumulation of Tregs might be associated with the development of ICI‐induced TIN.

ICIs have shown great promise for use in cancer treatment, but they have also resulted in a unique set of adverse immune reactions, classified as irAEs. Moreover, ICIs enhance tumor‐specific T cell responses, as well as nonspecific T cell activation.^11^ Two signals are required for T cell activation: antigen recognition and costimulation.^12^ During antigen recognition, T cells are activated by antigens presented by antigen‐presenting cells, via T cell receptors, but this is insufficient for full T cell activation. Upon costimulation, the costimulatory molecule (CD28) on the T cell surface binds to the shared ligand B7 (CD80/CD86). T cell activation is then maintained by activation of both the signals. Next, the PD‐1/PD‐L1 and CTLA‐4/B7 (CD80/CD86) pathways are involved in T cell suppression. T cell activation is suppressed by the binding of PD‐1 to PD‐L1, expressed on many cells, such as tumor and inflammatory cells, after cytokine exposure.^13^ Furthermore, the binding of B7 to CTLA‐4 instead of CD28 prevents costimulation and results in inhibition of T cell activation and proliferation.^14^

Monoclonal antibodies targeting PD‐1 or PD‐L1 block the PD‐1/PD‐L1 pathway and enhance T cell activation in both the tumor microenvironment and organs affected by irAEs.^2^, ^15^ Moreover, monoclonal antibodies targeting CTLA‐4 block inhibitory signals of the CTLA‐4/B7 (CD80/CD86) pathway on T cells, including Tregs, and suppress the inhibitory immune response.^3^ Therefore, T cell activation can be closely related to the development of irAEs.

Several ICI‐induced TIN case reports have shown that T cell infiltration is prominent in the tubulointerstitium. However, there is no consensus on whether CD4^+^ or CD8^+^ T cells are more significant for this. Furthermore, the detailed pathology of ICI‐induced TIN has not yet been demonstrated using immunohistochemistry owing mainly to low number of renal biopsies because acute kidney injury (AKI) is relatively uncommon among renal irAEs, with an incidence of 2%–5%.^5^, ^16^, ^17^, ^18^, ^19^ Therefore, we established baseline laboratory data and immunohistochemically compared the clinicopathological features of ICI‐induced TIN and non‐ICI drug‐induced TIN.

With respect to the baseline laboratory data, regardless of whether ICI or non‐ICI treatment was used, drug‐induced TIN was associated with high levels of sCr, consistent with previous research.^20^ Each patient with ICI‐induced TIN had a malignant tumor, and this positively correlated with age and CRP levels. However, there were no significant differences in urine NAG and β2MG levels between the two groups. Pathologically, ICI‐induced TIN showed less inflammatory cell infiltration in the renal interstitium compared with that in non‐ICI drug‐induced TIN, where T cells and macrophages comprised the primary cells, with very few other inflammatory cells, including B cells, plasma cells, neutrophils, and eosinophils. Hultin et al.^21^ reported that interstitial inflammation, ranging from mild to severe, occurs in ICI‐induced TIN. We found that the numbers of T cells, plasma cells, neutrophils, and eosinophils are useful for differentiating ICI‐induced from non‐ICI drug‐induced TIN. The number of B cells, especially in highly active inflammatory areas, is also useful in their differentiation.

We attempted to elucidate the mechanism of ICI‐induced TIN by comparing the pathological features of ICI‐induced and non‐ICI drug‐induced TIN using immunostaining. Although the number of CD4^+^ cells per square millimeter in ICI‐induced TIN was significantly lower than that in non‐ICI drug‐induced TIN, the number of CD8^+^ cells per square millimeter did not differ significantly between the two groups. However, the CD8/CD3 and CD8/CD4 ratios in ICI‐induced TIN were higher than those in non‐ICI drug‐induced TIN, suggesting that CD8^+^ cells are the more predominant T cell subsets in ICI‐induced TIN. Sobol et al.^22^ reported that ICI‐related myocarditis is characterized by CD8^+^ T cell infiltration. In addition, ICI‐induced colitis has been reported to be associated with marked CD8^+^ cell infiltration and a high CD8/CD4 ratio, distinguishing it from other forms of colitis, including ulcerative colitis, Crohn's disease, and ischemic colitis. ICI‐induced TIN is also characterized by a predominant CD8^+^ cell distribution, which distinguishes it from non‐ICI drug‐induced TIN.

Fewer CD25^+^ and FOXP3^+^ cells, which are characteristic markers of Tregs, were observed in ICI‐induced TIN than in non‐ICI drug‐induced TIN. Some reports have indicated that number of Tregs might decrease following inhibition of CTLA4 and PDL1 signaling. Amarnath et al.^23^ reported that the differentiation of human CD4^+^ cells to a tolerogenic Treg phenotype is promoted by PD‐L1 in vivo in a human‐into‐mouse xenogeneic graft‐versus‐host disease model. In addition, Romano et al.^24^ showed that FcγRIIIA (CD16)‐expressing nonclassical monocytes engaged by ipilimumab destroy Tregs ex vivo via antibody‐dependent cell‐mediated cytotoxic effects. Therefore, blocking either the PD‐1/PD‐L1 pathway or the CTLA‐4/B7 (CD80/CD86) pathway decreases the number of Tregs. Mihic‐Probst et al.^25^ also reported that CD25^+^ cells were sparse or absent in the autopsied organs of all patients with irAEs, except in the thyroid and pituitary glands of one patient, indicating low accumulation of Tregs with ICI treatment.

ICI‐induced TIN is characterized by localized inflammation. Even when the analysis was focused on areas exhibiting active inflammation, the predominant distribution of CD8^+^ cells and low accumulation of FOXP3^+^ cells were also observed in ICI‐induced TIN compared with that in non‐ICI drug‐induced TIN. Moreover, comparison of active and nonactive inflammatory areas in ICI‐induced TIN showed the same predominant distribution of CD8^+^ cells and the same ratio of FOXP3 to CD4 and CD8. These findings suggest that a similar mechanism might be responsible for tubulointerstitial inflammation in ICI‐induced TIN regardless of the tubulointerstitial inflammatory activity. The association between FOXP3^+^ and CD8^+^ cells might contribute to tubulointerstitial inflammation in ICI‐induced TIN, supported by the lower FOXP3/CD8 ratio of ICI‐induced TIN than that in non‐ICI drug‐induced TIN. Hagiwara et al.^26^ showed accumulation of CD8^+^ T cells and impaired activation of FOXP3^+^ cells in patients with hepatic irAEs. Additionally, in a genetic mouse model of ICI‐related myocarditis, inflammatory cells observed in the heart comprised CD8^+^ T cells and a small number of CD4^+^ and FOXP3^+^ T cells.^27^ These findings suggest that inadequate accumulation of Tregs might reflect significant CD8^+^ cell distribution. It is possible that ICIs decreased the Treg population and resulted in predominant CD8^+^ cell infiltration.

Different degrees of tublointerstitial inflammation can be seen in ICI‐induced TIN, which might occur due to loss of tolerance to endogenous antigens in the respective regions of the renal cortex.^5^ This notion is supported by the uniformity in the distribution of inflammatory cells in ICI‐induced TIN and the absence of ICI‐specific T cells in the peripheral blood.^28^ Contrarily, in non‐ICI drug‐induced TIN, drug‐specific T cells could release cytokines and cause subsequent tubulointerstitial injury,^29^ which results in diffuse inflammatory cell infiltration in non‐ICI drug‐induced TIN.

This study has several limitations, including the relatively low incidence of renal irAEs and assessment of the absolute number of inflammatory cells in ICI‐induced and non‐ICI drug‐induced TIN, which is different from nonpharmacological idiopathic TIN. The study was retrospective and included a few cases; thus, the interpretation of the histopathological features of ICI‐induced TIN was limited. Moreover, combination chemotherapy, in addition to ICIs, was commenced in some patients with ICI‐induced TIN, and this could have affected the kidney biopsy. In addition, renal biopsies show only snapshots of the consequences of injury and repair, making it challenging to identify the mechanism of ICI‐induced TIN.

In conclusion, we demonstrate the histopathological features of ICI‐induced TIN, compared with those of non‐ICI drug‐induced TIN, using detailed immunohistochemical staining. The primary inflammatory cells in ICI‐induced TIN were found to be T cells and macrophages, with few B cells, plasma cells, neutrophils, and eosinophils. An assessment of the numbers of T cells, plasma cells, neutrophils, and eosinophils could be beneficial to rule out non‐ICI drug‐induced TIN. The number of B cells in ICI‐induced TIN could also help rule out non‐ICI drug‐induced TIN, especially in active inflammatory areas. Compared with non‐ICI drug‐induced TIN, ICI‐induced TIN showed lower accumulation of Tregs and a predominant CD8^+^ cell distribution among T cells, regardless of the tubulointerstitial inflammatory activity. Moreover, the loss of tolerance to endogenous renal antigens might be caused by this low accumulation of Tregs and predominant CD8^+^ cell distribution. Our findings show the distinction between ICI‐induced and non‐ICI drug‐induced TIN and provide important insights into the development of ICI‐induced TIN. Further investigations are required to confirm our findings by comparing ICI‐induced TIN with non‐drug idiopathic TIN and to develop more specific treatments for ICI‐induced TIN.

## AUTHOR CONTRIBUTIONS

Kenta Tominaga designed the experiments, and Kenta Tominaga and Etsuko Toda analyzed and interpreted the patient data in the context of kidney disease under the supervision of Akira Shimizu. All authors read and approved the final manuscript.

## CONFLICT OF INTEREST STATEMENT

Akira Shimuzu and Yasuhiro Terasaki is an Editorial Board member of Pathology International and co‐authors of this article. To minimize bias, they were excluded from all editorial decision‐making related to the acceptance of this article for publication.

## Supporting information

Supporting information.

Supporting information.

Supporting information.

Supporting information.

## Data Availability

The data are available from the corresponding author (ashimizu@nms.ac.jp) upon reasonable request.
