# Supplementary figures and images for: Predominant CD8+ cell infiltration and low accumulation of regulatory T cells in immune checkpoint inhibitor‐induced tubulointerstitial nephritis
Source: Pathol Int. 2024 Apr 18;74(6):317–26. doi: 10.1111/pin.13428 (PMC11551812; doi:10.1111/pin.13428)

## Slide 1
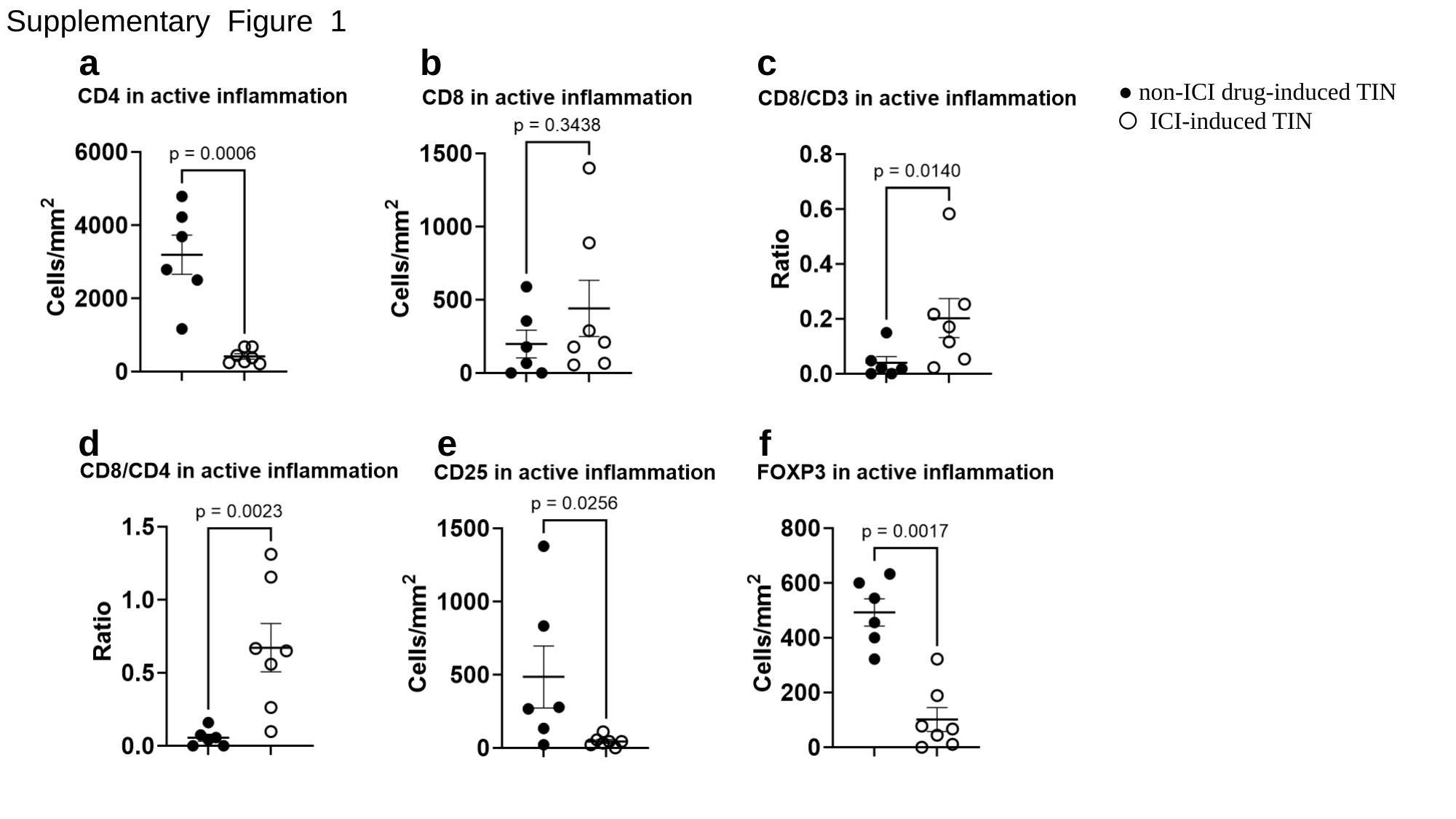

Supplementary Figure 1
a
b
c
● non-ICI drug-induced TIN
〇 ICI-induced TIN
e
f
d

Supplement: Supplementary file 1 — Supporting information. [file PIN-74-317-s004.pptx]
