# Supplementary material for: Predominant CD8+ cell infiltration and low accumulation of regulatory T cells in immune checkpoint inhibitor‐induced tubulointerstitial nephritis
Source: Pathol Int. 2024 Apr 18;74(6):317–26. doi: 10.1111/pin.13428 (PMC11551812; doi:10.1111/pin.13428)
Supplement: Supplementary file 2 — Supporting information. [file PIN-74-317-s002.pptx]

## Slide 1
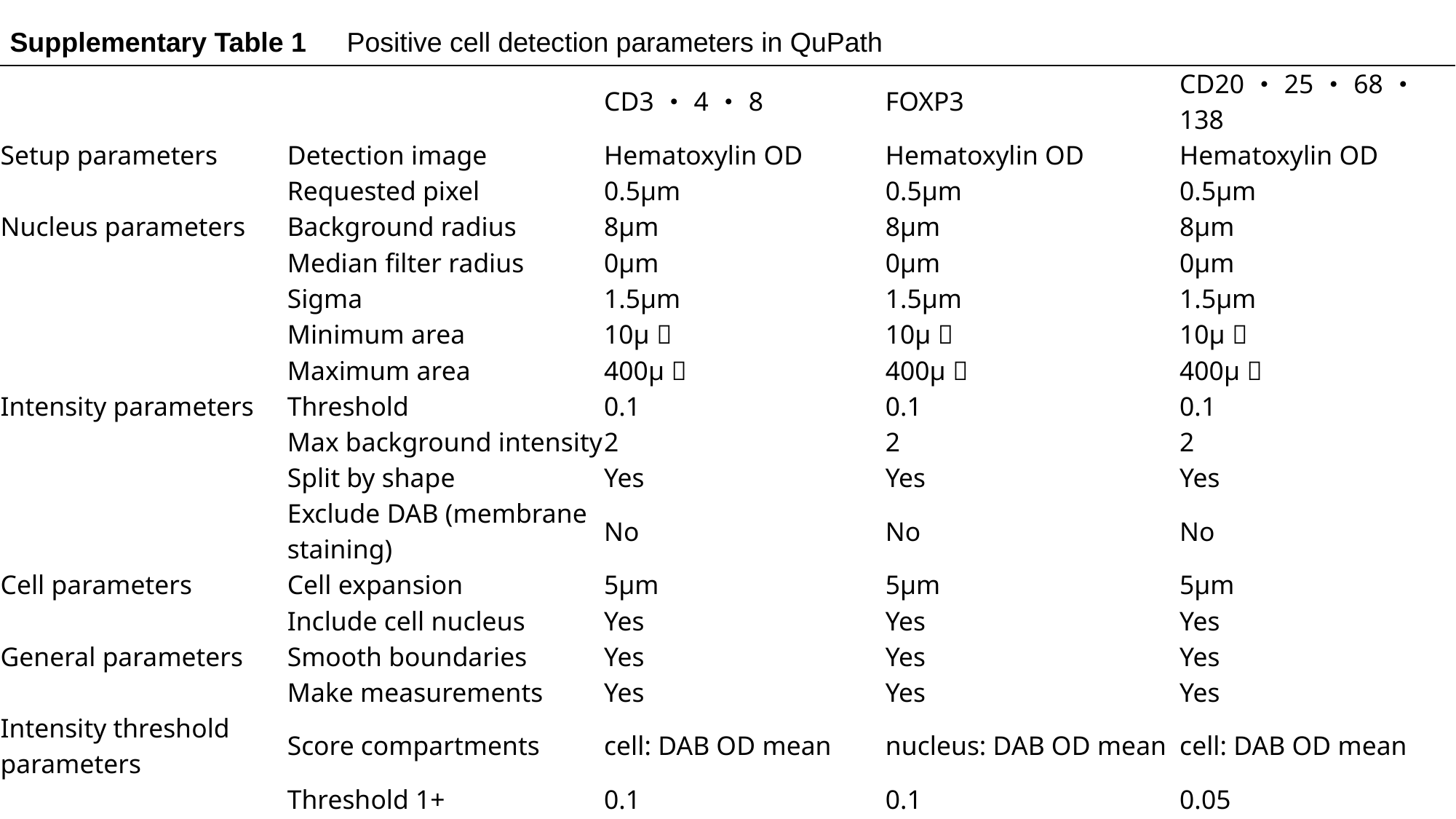

Supplementary Table 1　Positive cell detection parameters in QuPath
| | | CD3・4・8 | FOXP3 | CD20・25・68・138 |
| --- | --- | --- | --- | --- |
| Setup parameters | Detection image | Hematoxylin OD | Hematoxylin OD | Hematoxylin OD |
| | Requested pixel | 0.5μm | 0.5μm | 0.5μm |
| Nucleus parameters | Background radius | 8μm | 8μm | 8μm |
| | Median filter radius | 0μm | 0μm | 0μm |
| | Sigma | 1.5μm | 1.5μm | 1.5μm |
| | Minimum area | 10μ㎡ | 10μ㎡ | 10μ㎡ |
| | Maximum area | 400μ㎡ | 400μ㎡ | 400μ㎡ |
| Intensity parameters | Threshold | 0.1 | 0.1 | 0.1 |
| | Max background intensity | 2 | 2 | 2 |
| | Split by shape | Yes | Yes | Yes |
| | Exclude DAB (membrane staining) | No | No | No |
| Cell parameters | Cell expansion | 5μm | 5μm | 5μm |
| | Include cell nucleus | Yes | Yes | Yes |
| General parameters | Smooth boundaries | Yes | Yes | Yes |
| | Make measurements | Yes | Yes | Yes |
| Intensity threshold parameters | Score compartments | cell: DAB OD mean | nucleus: DAB OD mean | cell: DAB OD mean |
| | Threshold 1+ | 0.1 | 0.1 | 0.05 |
| | Threshold 2+ | 0.4 | 0.4 | 0.4 |
| | Threshold 3+ | 0.6 | 0.6 | 0.6 |
| | Single threshold | Yes | Yes | Yes |
