# Supplementary material for: Predominant CD8+ cell infiltration and low accumulation of regulatory T cells in immune checkpoint inhibitor‐induced tubulointerstitial nephritis
Source: Pathol Int. 2024 Apr 18;74(6):317–26. doi: 10.1111/pin.13428 (PMC11551812; doi:10.1111/pin.13428)
Supplement: Supplementary file 3 — Supporting information. [file PIN-74-317-s001.docx]

**Supplementary Table 2** Pathological characteristics of inflammatory cell infiltration in active inflammatory areas

|  | ICI | Non-ICI | P-value |
| --- | --- | --- | --- |
| **Interstitial inflammatory cell counts**  **per mm^2^** |  |  |  |
| T cells (CD3+ cells) | 2137 ± 460* | 5493 ± 917 | 0.014 |
| B cells (CD20+ cells) | 206 ± 88* | 769 ± 196 | 0.035 |
| Macrophages (CD68+ cells) | 1948 ± 747 | 3705 ± 694 | 0.1014 |
| Plasma cells (CD138+ cells) | 632 ± 399* | 2200 ± 729 | 0.0198 |
| Neutrophils | 10 ± 5* | 41 ± 8 | 0.0111 |
| Eosinophils | 11 ± 6* | 98 ± 49 | 0.0087 |

TIN: Tubulointerstitial nephritis, ICI: Immune checkpoint inhibitors * compared to non-ICI; p < 0.05
