# Supplementary material for: Predominant CD8+ cell infiltration and low accumulation of regulatory T cells in immune checkpoint inhibitor‐induced tubulointerstitial nephritis
Source: Pathol Int. 2024 Apr 18;74(6):317–26. doi: 10.1111/pin.13428 (PMC11551812; doi:10.1111/pin.13428)
Supplement: Supplementary file 4 — Supporting information. [file PIN-74-317-s003.docx]

**Supplementary Table 3** T cell distribution in nonactive inflammatory areas of ICI-induced TIN compared with that in active inflammatory areas of ICI-induced TIN and non-ICI drug-induced TIN

|  | Nonactive area in ICI | Active area in ICI | Non-ICI | P-value (Nonactive area vs Active area in ICI) | P-value (Nonactive area in ICI vs Non-ICI) | P-value (active area in ICI vs Non-ICI) |
| --- | --- | --- | --- | --- | --- | --- |
| CD8/CD3 ratio | 0.2±0.1 | 0.2±0.1 | 0.05±0.02 | >0.9999 | 0.0513 | 0.035 |
| CD8/CD4 ratio | 1.4±0.9 | 0.7±0.2 | 0.09±0.03 | 0.9015 | 0.1807 | 0.0047 |
| FOXP3/CD4 ratio | 0.5±0.4 | 0.2±0.04 | 0.2±0.02 | >0.9999 | 0.7308 | 0.9452 |
| FOXP3/CD8 ratio | 0.4±0.1 | 0.3±0.1 | 4.2±2.0 | 0.2721 | 0.0023 | 0.0012 |

TIN: Tubulointerstitial nephritis, ICI: Immune checkpoint inhibitors
